# Development of a Novel Automated Workflow in Fiji ImageJ for Batch Analysis of Confocal Imaging Data to Quantify Protein Colocalization Using Manders Coefficient

**DOI:** 10.21769/BioProtoc.5285

**Published:** 2025-04-05

**Authors:** Vikram Aditya, Vishakha Tambe, Wei Yue

**Affiliations:** Department of Pharmaceutical Sciences, University of Oklahoma Health Sciences Center, Oklahoma City, OK, USA;

**Keywords:** Confocal microscopy, Colocalization, Manders coefficient, Fiji ImageJ, BIOP-JACoP, Auto-thresholding, Automation

## Abstract

Confocal microscopy is integral to molecular and cellular biology, enabling high-resolution imaging and colocalization studies to elucidate biomolecular interactions in cells. Despite its utility, challenges in handling large datasets, particularly in preprocessing Z-stacks and calculating colocalization metrics like the Manders coefficient, limit efficiency and reproducibility. Manually processing large numbers of imaging data for colocalization analysis is prone to observer bias and inefficiencies. This study presents an automated workflow integrating Python-based preprocessing with Fiji ImageJ's BIOP-JACoP plugin to streamline Z-stack refinement and colocalization analysis. We generated an executable Windows application and made it publicly available on GitHub (https://github.com/weiyue99/Yue-Colocalization), allowing even those without Python experience to directly run the Python code required in the current protocol. The workflow systematically removes signal-free Z-slices that sometimes exist at the beginning and/or end of the Z-stacks using auto-thresholding, creates refined substacks, and performs batch analysis to calculate the Manders coefficient. It is designed for high-throughput applications, significantly reducing human error and hands-on time. By ensuring reproducibility and adaptability, this protocol addresses critical gaps in confocal image analysis workflows, facilitating efficient handling of large datasets and offering broad applicability in protein colocalization studies.

Key features

• Automated image analysis: use of Python-based code for substack creation based on auto-thresholding to eliminate observer bias.

• Manders coefficient calculation: quantification of colocalization using BIOP-JACoP in Fiji ImageJ.

• High-throughput compatibility: efficient for large datasets using macro codes to run in batch mode; minimal manual intervention.

## Graphical overview


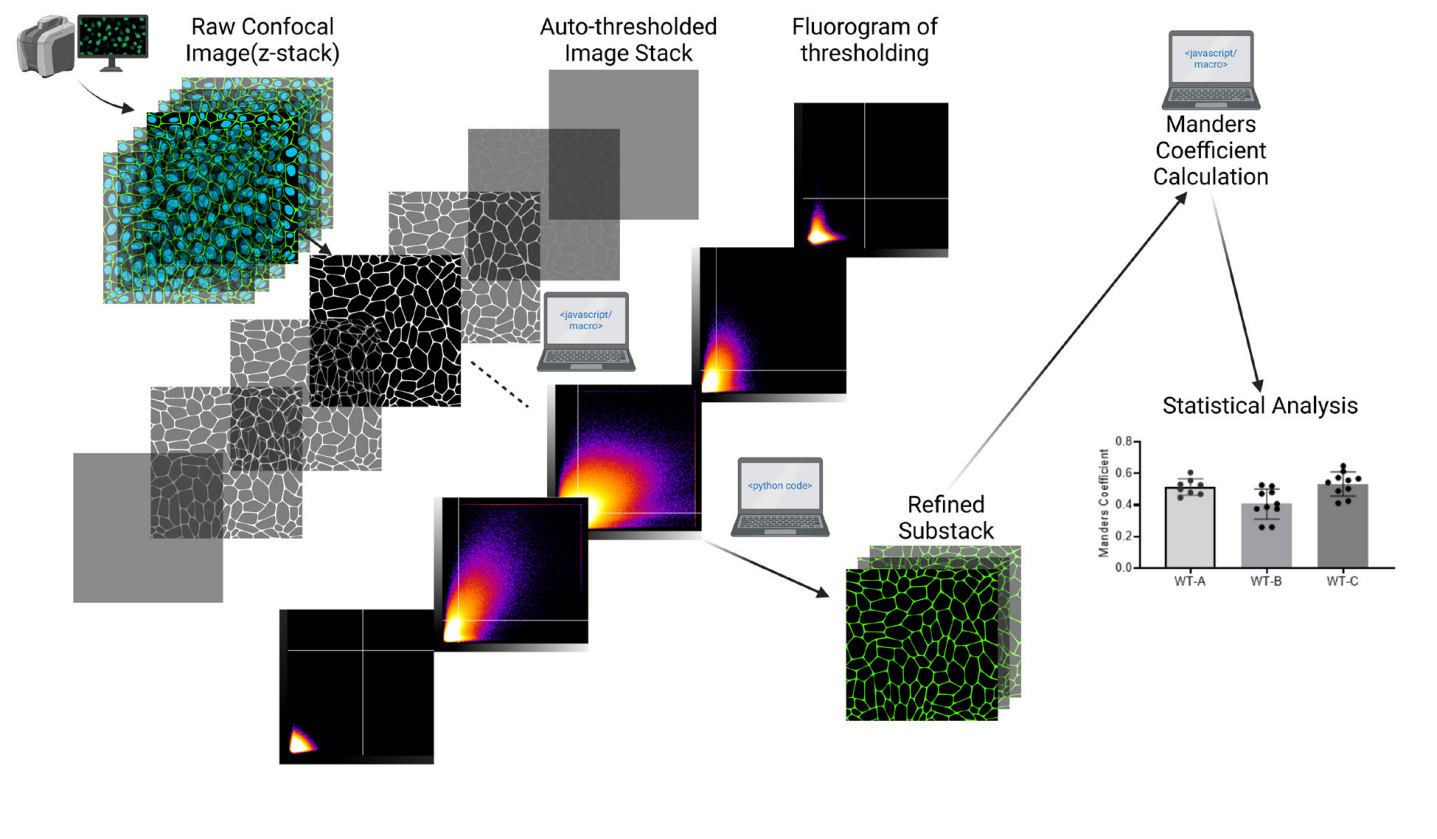


Created in BioRender. Aditya, V. (2025) https://BioRender.com/m89i580

## Background

Confocal microscopy is a cornerstone of modern molecular and cell biology research, providing high-resolution imaging capabilities and generating large volumes of data, especially when performing Z-stack imaging. For example, systems like the Olympus FV10i-LIV or Zeiss LSM series can automatically produce hundreds of images per sample, enabling detailed spatial and temporal studies of biological specimens [1,2]. Among the many applications of confocal microscopy, colocalization analysis is a widely used technique to assess the spatial association between biomolecules, helping to infer protein interactions, co-occurrence, and functional relationships [3]. The Manders coefficient is a commonly employed metric for colocalization studies, offering a quantitative measure of overlap between two signals in fluorescence microscopy datasets [4]. Fiji ImageJ’s Bioimaging and Optics Platform (BIOP) Just Another Colocalization Plugin (JACoP) has been developed for colocalization analysis on confocal images by using the Manders coefficient method [5,6].

Despite its importance, conducting accurate and efficient colocalization analysis remains challenging due to the lack of automated workflows to handle the substantial datasets generated by confocal microscopy. To the best of our knowledge, no comprehensive, automated solution exists in the literature to address these issues, creating an urgent and unmet need for a step-by-step protocol for automated workflows. The primary challenge lies in the preprocessing of Z-stack images, which often include blank slices at the beginning and end of the stack due to the nature of sample preparation and imaging conditions. Including these signal-free slices in Manders coefficient calculations may disrupt thresholding accuracy and lead to inconsistent results [3]. Manually identifying and excluding these blank images is not only time-consuming but also prone to observer bias, which can introduce errors and inconsistencies across datasets.

The current protocol addresses these limitations by providing a detailed, step-by-step automated workflow that integrates a Python-based script to refine raw confocal images. The script identifies and removes signal-free Z-slices, creating refined substacks for further analysis. By coupling this preprocessing step with Fiji ImageJ’s BIOP-JACoP plugin for Manders coefficient calculations, the protocol ensures reliable and reproducible colocalization measurements. This automated approach minimizes human intervention, enhances the accuracy of results, and significantly improves the efficiency of high-throughput studies.

Organic anion transport polypeptide (OATP) 1B1 is a plasma membrane transport protein that plays an important role in drug disposition [7]. Plasma membrane localization of OATP1B1 has often been assessed by quantification of its colocalization with sodium-potassium ATPase (Na/K-ATPase), a plasma membrane protein marker, as we previously published [8,9]. The current protocol uses colocalization of transiently transfected FLAG-tagged OATP1B1 with endogenous Na/K-ATPase in human embryonic kidney (HEK) 293 cells from confocal images as an example, as shown in our original publication [9].

## Materials and reagents

**Biological materials**

1. HEK293 cells (American Type Culture Collection, CRL-1573)

2. pcDNA3.1(+)-WT-OATP1B1: pcDNA3.1 (+) vector-based mammalian expression plasmid vector with the open reading frame of wild-type (WT) OATP1B1 was subcloned at GenScript Biotech (Piscataway, NJ) and verified by sequencing, as described in our publication [9]

**Reagents**

1. Lipofectamine 2000 transfection reagent (Thermo Fisher Scientific, catalog number: 11668019)

2. Antibodies

Primary antibodies: Rabbit FLAG polyclonal antibody (Sigma-Aldrich, catalog number: F7425) and mouse monoclonal Na/K-ATPase antibody (Santa Cruz, catalog number: sc-21712)

Secondary antibodies: Alexa Fluor 594 goat anti-rabbit IgG (Thermo Fisher Scientific, catalog number: A-11012) and Alexa Fluor^®^ 488 goat anti-mouse IgG antibody (Thermo Fisher Scientific, catalog number: A-11001)

3. 4’,6-diamidino-2-phenylindole, dihydrochloride (DAPI) (Thermo Fisher Scientific, catalog number: D1306)

## Equipment

1. Fluoview microscope (Olympus, model: FV10i-LIV Confocal Imaging System with Live-Cell)

2. Computer system (Windows 10 Enterprise, Processor: 12th Gen Intel^®^ Core^TM^ i5-12500, 3.00 GHz, RAM: 16.0 GB)

*Note: Any computer system capable of running Fiji ImageJ on supported platforms is acceptable.*

## Software and datasets

1. Fiji ImageJ (1.54f, 09/15/2024) [6], open source (https://imagej.net/Fiji/Downloads)

2. BIOP-JACoP (v2.1.1, 08/20/2010) installed to Fiji ImageJ:

Download JACoP from https://github.com/fabricecordelieres/IJ-Plugin_JACoP/releases/download/v2.1.4/JACoP_.jar. Launch Fiji, *Help* → *Update → manage update sites.* In the *search* box, type in PTBIOP *→ Apply and close → Apply changes → Close.*

3. Python (v3.12.6, 09/06/2024), open source (https://www.python.org/downloads/)

4. GraphPad Prism (10.4.1, 12/05/2024), requires subscription (RRID:SCR_002798, https://www.graphpad.com)

4. All code and executable Windows applications have been deposited to GitHub and are publicly available: https://github.com/weiyue99/Yue-Colocalization (10/4/2024).

## Procedure

The current protocol provides a detailed step-by-step procedure for conducting batch analysis to determine the Manders coefficient for colocalization quantification using confocal z-stack datasets from immunofluorescent staining. A detailed protocol for immunofluorescent staining and confocal imaging has been described in our previous publications [8,9]. In brief, HEK293 cells were transiently transfected with pcDNA3.1(+)-WT-OATP1B1 using Lipofectamine following the manufacturer’s instructions. Forty-eight hours after transfection, immunofluorescent staining was conducted using rabbit anti-FLAG (1:100 dilution) and mouse anti-Na/K-ATPase (1:50 dilution) antibodies [8,9] and detected by Alexa Fluor 594 and Alexa Fluor 488 conjugated goat anti-rabbit and goat-anti-mouse secondary antibodies, respectively. Nuclei were counter-stained by DAPI.

**Figure 1.**
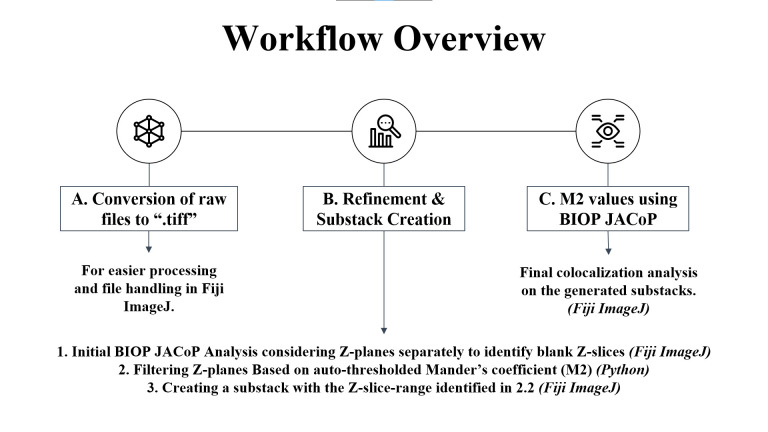
Workflow overview showing the key steps of the protocol. (A) Raw files are first converted to .tiff format in Fiji ImageJ. (B) Python code and Fiji ImageJ macro are utilized to refine and create substack images suitable for subsequent colocalization analysis in (C) to determine M2 values using the Manders coefficient method in Fiji ImageJ via the BIOP JACoP plugin.

Confocal microscopy was conducted using an FV10i-LIV confocal microscope. Imaging was performed with excitation wavelengths of 405 nm (DAPI), 473 nm (Alexa Fluor 488), and 559 nm (Alexa Fluor 594). High-resolution images were captured at a matrix size of 1,024 × 1,024 pixels, with a pixel size of 0.207 μm and Z-slices spaced at 1 µm intervals, as described in our previous publication [9]. The current protocol uses WT-OATP1B1 data as an example (original Figure 8, WT in panels A–C from [9]). Signals of DAPI, Na/K-ATPase, and FLAG were designated to channels C1, C2, and C3, respectively. The extent of colocalization of FLAG-tagged OATP1B1 with Na/K-ATPase was determined by the Manders coefficient, which is the fraction of FLAG overlapping with Na/K-ATPase denoted as M2 in Image J output and used consistently throughout this manuscript. A brief overview of the current protocol is shown in Figure 1, followed by detailed step-by-step descriptions of each step.

**A. Conversion of raw confocal files to .tiff files using Fiji ImageJ’s batch convert**

The raw image files generated from confocal microscopy must be converted to .tiff format to enable compatibility with downstream analysis tools. This conversion is essential as the .tiff format is universally supported by image processing software, including Fiji ImageJ[6], and can be easily read and processed using JavaScript-based tools. The .tiff format ensures efficient handling of large image datasets while maintaining image fidelity and metadata.

Use the *Batch Convert* function in Fiji ImageJ for this process and organize the resulting .tiff images into a dedicated directory, preparing them for subsequent steps in the workflow.

**Figure 2.**
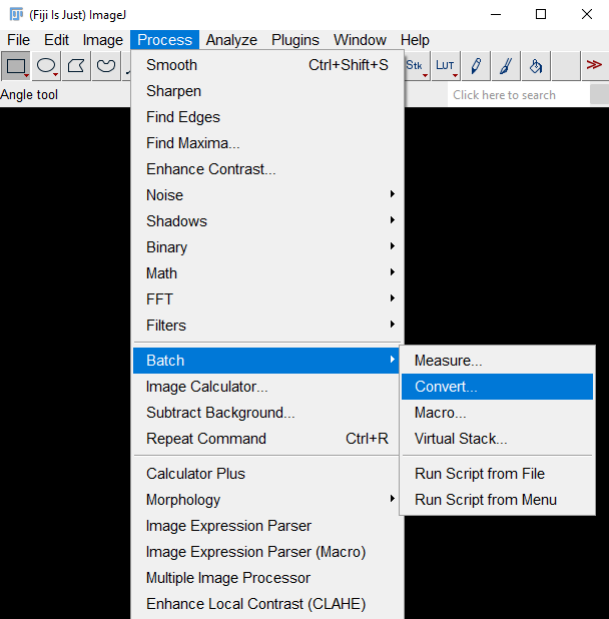
Screenshot of navigation through Fiji ImageJ to access the *Batch Convert* tool

**Figure 3.**
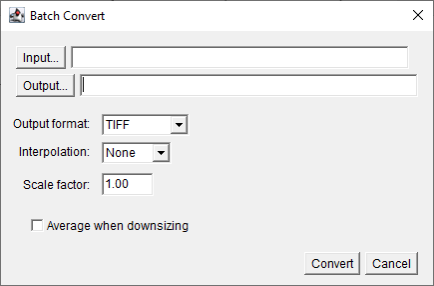
Screenshot of *Batch Convert* tool interface. Choose Input and Output file directory.

1. Open *Fiji ImageJ.*

2. Go to *Process → Batch →Convert…* (Figure 2).

3. Select the folder containing the raw confocal image files in *Input…* and the folder where the .tiff files should be saved as *Output…* (Figure 3).

4. Interpolation: *None.*

5. Click *Convert*.

**B. Image refinement and substack creation**

Often, z-slices near the beginning and end of the z-stack have no fluorescent signal if these border z-slices are out of the range of thickness of the cells. In such z-slices, thresholded M2 values are reported as 0 or null (NaN) from the output of the BIOP JACoP. Follow the steps below to refine the Z-stack, creating a substack without slices containing 0 or null M2 values:

1. Initial BIOP JACoP batch Analysis in Fiji ImageJ to identify and locate blank Z-slices: Perform an initial auto-thresholding batch analysis using the BIOP JACoP plugin to determine Manders coefficients (M2), considering Z-planes separately. This analysis shows thresholded signals from each plane. The manual steps described below describe how to perform such analysis on one image file at a time. To process multiple images simultaneously, users should skip the *Manual steps* and directly proceed to the *Batch analysis* below.

**Figure 4.**
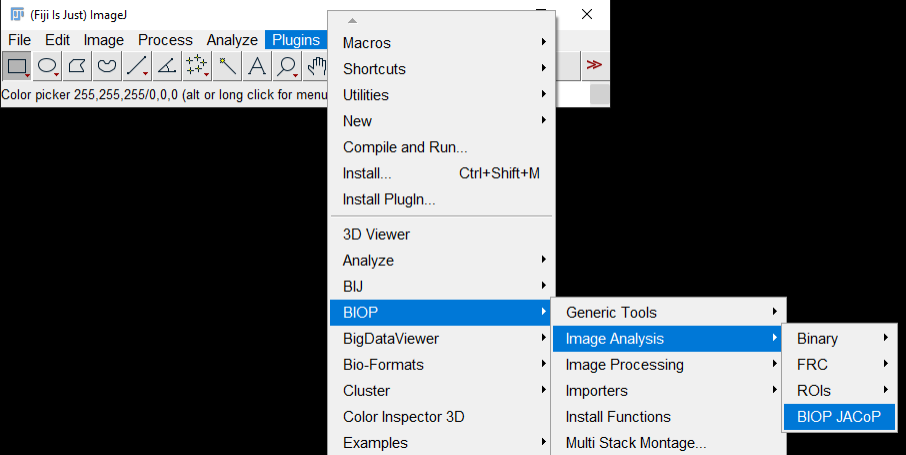
Screenshot of navigation through Fiji ImageJ to access the BIOP JACoP plugin

**Figure 5.**
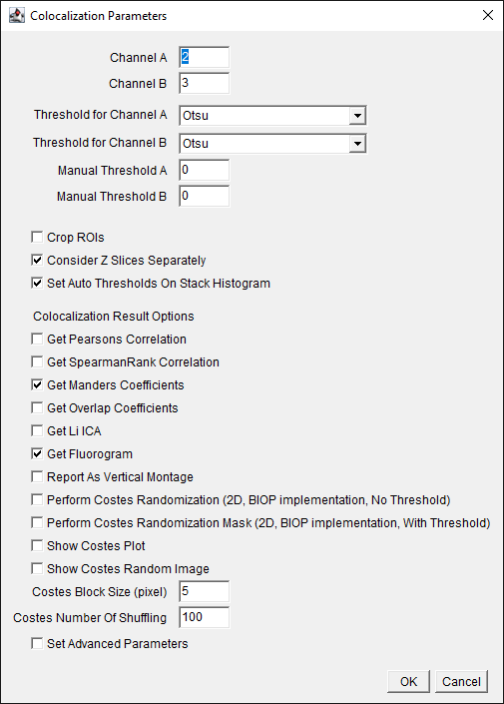
Screenshot of BIOP JACoP plugin’s *Colocalization Parameters* interface using Manders coefficient. Choose channels where the signal of interest is present. Choose the auto-thresholding (Otsu) method for each channel from the drop-down menu for *Threshold for Channel*. Check the boxes for options *Consider Z Slices Separately, Set Auto Thresholds On Stack Histogram, Get Manders Coefficients*, and *Get Fluorogram*.

*Note: Our current protocol focuses on using the Manders Coefficient method [4] for colocalization determination. Using other methods listed on this interface for colocalization analysis is beyond the scope of our protocol. Users can refer to the BIOP-JACoP readme file* *https://imagej.net/plugins/coloc-2* *and some original citations [11,12] for more information about other methods listed on this interface.*

Manual steps:

a. Open Fiji and load the raw image for analysis.

b. Go to *Plugins → BIOP → Image Analysis → BIOP JACoP* (Figure 4).

c. Choose the channel of interest for colocalization (Figure 5). For our dataset, assign Channel A as 2 (C2), which is the Na/K-ATPase signal, and assign Channel B as 3 (C3), which is the FLAG-OATP1B1 signal. The thresholding method is Otsu for both channels. Check to *Consider Z-slices Separately*, and select *OK*.

*Note: Otsu is one of the auto-thresholding methods. The current protocol uses the commonly used Otsu [10] as an example. Refer to General note 1 for further optimizations.*

d. Save the results as a .csv file in *File → Save as*.

**Figure 6.**
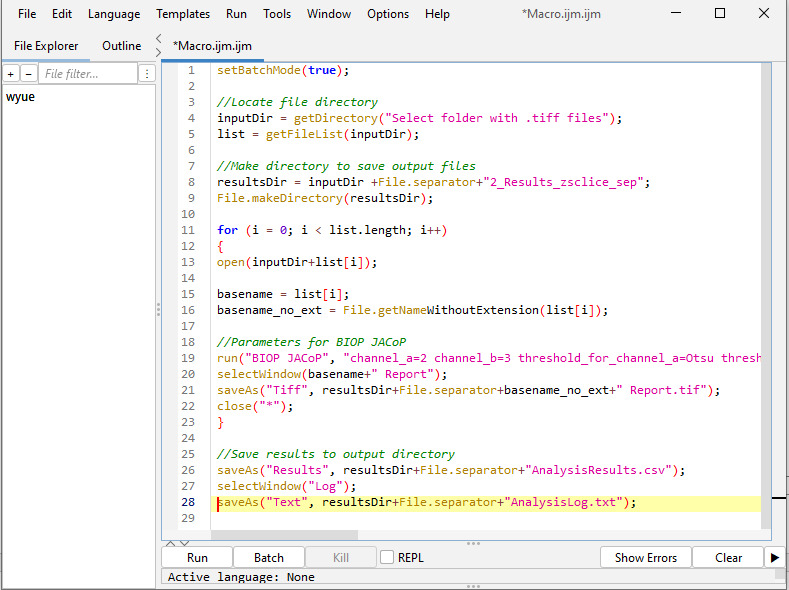
Screenshot of B.1 macro code in Fiji ImageJ

Batch analysis: Run the above manual steps process in batch mode using the following macro code in Fiji ImageJ. (Refer to General note 2 for how to run a macro code in Fiji ImageJ.) A screenshot is provided in Figure 6 after inputting the below macro code in Fiji ImageJ.

*Note: This script analyzes all the image stacks in the root directory and generates the output in a single .csv file. This file contains thresholded Manders coefficient (M2) values for each Z-slice across all image stacks in the dataset. In the code, “//” denotes a comment in JavaScript.*

setBatchMode(true);

//Locate file directory

inputDir = getDirectory("Select folder with .tiff files");

list = getFileList(inputDir);

//Make directory to save output files

resultsDir = inputDir +File.separator+"2_Results_zsclice_sep";

File.makeDirectory(resultsDir);

for (i = 0; i < list.length; i++)

{

open(inputDir+list[i]);

basename = list[i];

basename_no_ext = File.getNameWithoutExtension(list[i]);

//Parameters for BIOP JACoP

run("BIOP JACoP", "channel_a=2 channel_b=3 threshold_for_channel_a=Otsu threshold_for_channel_b=Otsu manual_threshold_a=0 manual_threshold_b=0 consider_z_slices_separately set_auto_thresholds_on_stack_histogram get_manders get_fluorogram costes_block_size=5 costes_number_of_shuffling=100");

selectWindow(basename+" Report");

saveAs("Tiff", resultsDir+File.separator+basename_no_ext+" Report.tif");

close("*");

}

//Save results to output directory

**Figure 7.**
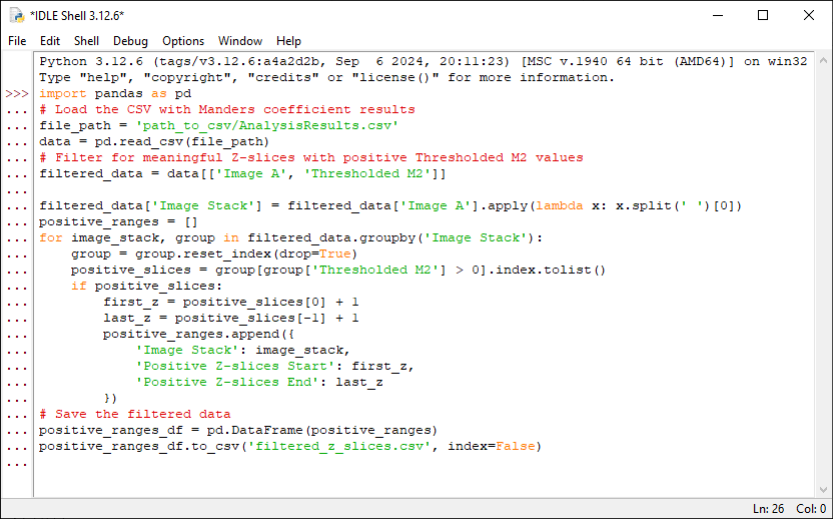
Screenshot of Python code in step B2 pasted in the Python editor as an example

*Note: To run this script, the user must have Python v3.7 or above with the pandas library available in their computer system.*

saveAs("Results", resultsDir+File.separator+"AnalysisResults.csv");

selectWindow("Log");

saveAs("Text", resultsDir+File.separator+"AnalysisLog.txt");

2. Find Z-planes with zero or null values using a Python script: Run the following Python script to identify ranges of positive M2 values, excluding null (NaN) or zero values. For each stack, it extracts the start and end Z-slice numbers corresponding to these positive ranges and generates a new .csv file that includes the image stack name and its Z-slice start and end numbers, facilitating precise substack creation for downstream analysis. A screenshot of Python code pasted in a text editor is provided in Figure 7. “#” denotes a comment in Python.

import pandas as pd

# Load the CSV with Manders coefficient results

file_path = 'path_to_csv/AnalysisResults.csv'

data = pd.read_csv(file_path)

# Filter for meaningful Z-slices with positive Thresholded M2 values

filtered_data = data[['Image A', 'Thresholded M2']]

filtered_data['Image Stack'] = filtered_data['Image A'].apply(lambda x: x.split(' ')[0])

positive_ranges = []

for image_stack, group in filtered_data.groupby('Image Stack'):

 group = group.reset_index(drop=True)

 positive_slices = group[group['Thresholded M2'] > 0].index.tolist()

 if positive_slices:

 first_z = positive_slices[0] + 1

 last_z = positive_slices[-1] + 1

 positive_ranges.append({

 'Image Stack': image_stack,

 'Positive Z-slices Start': first_z,

 'Positive Z-slices End': last_z

 })

# Save the filtered data

positive_ranges_df = pd.DataFrame(positive_ranges)

positive_ranges_df.to_csv('filtered_z_slices.csv', index=False)

**Figure 8.**
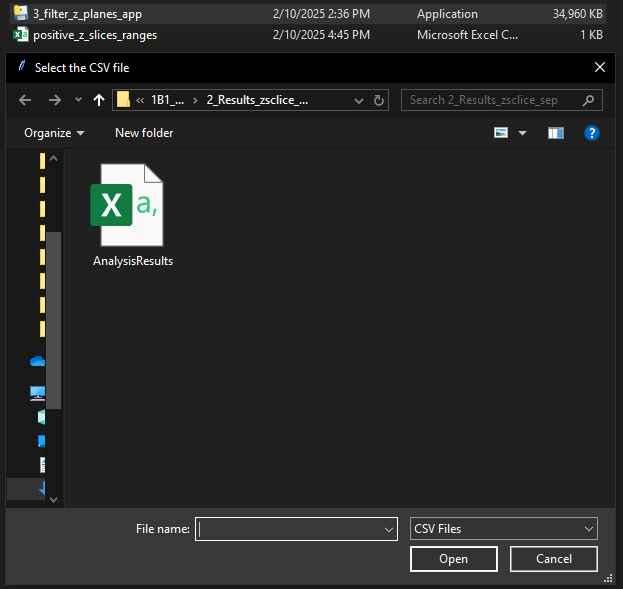
Screenshot of GUI dialog box that opens after executing the Python application (.exe) in Windows. Choose the *AnalysisResults.csv* file generated in Step B1 and click *Open.* The Python application will generate a *positive_z_slices_ranges.csv* file in the same directory where the .exe application is present.

To minimize software dependency, we generated an executable Windows application and made it publicly available on GitHub (https://github.com/weiyue99/Yue-Colocalization) (Figure 8), allowing even those without Python experience to directly run this Python code (see also *Troubleshooting*). To do so:

a. Download the *3_filter_z_planes_app.exe* from the above GitHub link. Right-click and *Save link as → Save.*

b. Double-click to run this application.

*Note: You need admin privileges on your computer to allow this application for the first time.*

c. A graphical user interface (GUI) dialog box will open (Figure 8). Select the .csv file generated in step B1 and click *Open.* The Python application will create a file *positive_z_slices_ranges.csv* in the same directory where the .exe application is present.

**Figure 9.**
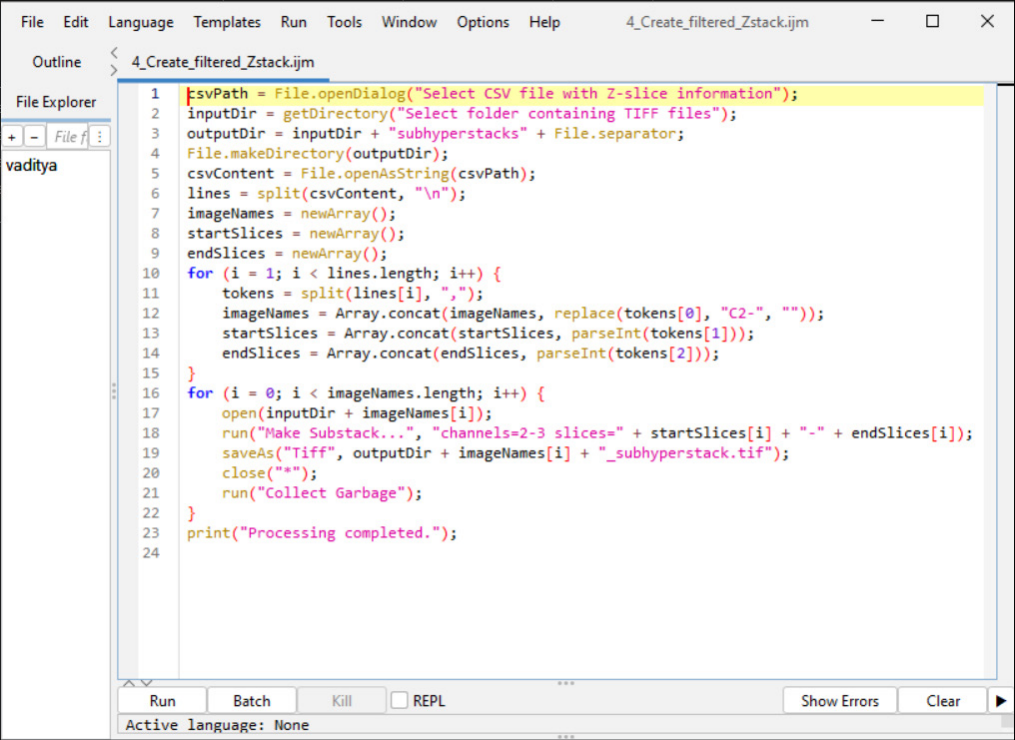
Screenshot of B3 macro code for substack creation in Fiji ImageJ

3. Substack creation in Fiji ImageJ: The *Make Substack...* function in Fiji ImageJ is used to generate a substack from the raw .tiff files created in Section A. Each substack includes only two relevant channels and is limited to the Z-slice range identified in step 2. A screenshot is provided in Figure 9 after inputting the below macro code in Fiji ImageJ.

a. Run the following macro. (Refer to General note 2 for how to run a macro code in Fiji ImageJ.)

//Locate csv file with z-slice information

csvPath = File.openDialog("Select CSV file with Z-slice information");

inputDir = getDirectory("Select folder containing TIFF files");

//Make a directory to save output file

outputDir = inputDir + "subhyperstacks" + File.separator;

File.makeDirectory(outputDir);

//Read the csv file, and collect relevant columns

csvContent = File.openAsString(csvPath);

lines = split(csvContent, "\n");

imageNames = newArray();

startSlices = newArray();

endSlices = newArray();

for (i = 1; i < lines.length; i++) {

 tokens = split(lines[i], ",");

 //removes the channel name to match with file name, and collect start & end

 imageNames = Array.concat(imageNames, replace(tokens[0], "C2-", ""));

 startSlices = Array.concat(startSlices, parseInt(tokens[1]));

 endSlices = Array.concat(endSlices, parseInt(tokens[2]));

}

//Make substack

for (i = 0; i < imageNames.length; i++) {

 open(inputDir + imageNames[i]);

 run("Make Substack...", "channels=2-3 slices=" + startSlices[i] + "-" + endSlices[i]);

 //Save file

 saveAs("Tiff", outputDir + imageNames[i] + "_subhyperstack.tif");

 close();

}

b. A dialog box will open. Select the *filtered_z_slices.csv* generated in step 2 and then select the folder containing the original .tiff files from Section A.

c. The macro will create new .tiff files containing two channels of interest with the filtered Z-planes.

**C. Manders coefficient analysis on filtered substacks using BIOP JACoP**

This step calculates the Manders coefficient (M2) to quantify the overlap between two signals of interest. In our case, this is Channel 2 (Na/K-ATPase) and Channel 3 (FLAG-OATP1B1) within the selected Z-slice range.

The “manual steps” below describe how to perform such analysis on one image file at a time. To process multiple images simultaneously, users should skip the *Manual steps* and directly proceed to the *Batch analysis* described below.

**Figure 10.**
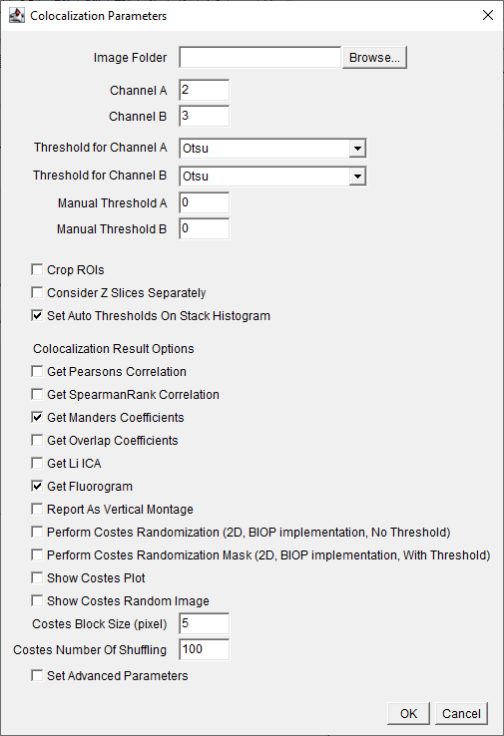
Screenshot of BIOP JACoP plugin’s *Colocalization Parameters* interface. Choose channels where the signal of interest is present. Choose the auto-thresholding method for each channel from the drop-down menu for *Threshold for Channel*. Check the boxes for options *Set Auto Thresholds On Stack Histogram, Get Manders Coefficients*, and *Get Fluorogram*.

Manual steps:

1. Open *Fiji* and load the filtered substack for analysis.

2. Go to *Plugins → BIOP → Image Analysis → BIOP JACoP* (Figure 4).

3. Choose the channel of interest for colocalization. Here, it was C2: Na/K-ATPase and C3: FLAG. Thresholding method is *Otsu*, **uncheck** *Consider Z slices Separately*, and select *OK* (Figure 10).

4. Save the results as a .csv file in *File* → *Save as*.

**Figure 11.**
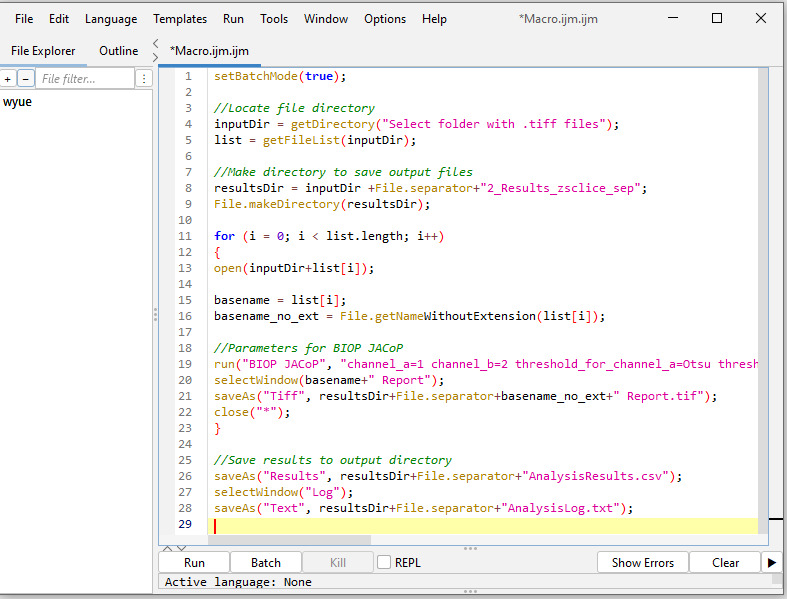
Screenshot of macro code of batch analysis from Section C in Fiji ImageJ

Batch analysis:

To reduce hands-on time, run this process in batch mode using the following macro code. (Refer to General note 2 for how to run macro code in Fiji ImageJ.) A screenshot is provided in Figure 11 after inputting the below macro code in Fiji ImageJ.

setBatchMode(true);

//Locate file directory

inputDir = getDirectory("Select folder with .tiff files");

list = getFileList(inputDir);

//Make directory to save output files

resultsDir = inputDir +File.separator+"2_Results_zsclice_sep";

File.makeDirectory(resultsDir);

for (i = 0; i < list.length; i++)

{

open(inputDir+list[i]);

basename = list[i];

basename_no_ext = File.getNameWithoutExtension(list[i]);

//Parameters for BIOP JACoP

run("BIOP JACoP", "channel_a=1 channel_b=2 threshold_for_channel_a=Otsu threshold_for_channel_b=Otsu manual_threshold_a=0 manual_threshold_b=0 set_auto_thresholds_on_stack_histogram get_manders get_fluorogram costes_block_size=5 costes_number_of_shuffling=100");

selectWindow(basename+" Report");

saveAs("Tiff", resultsDir+File.separator+basename_no_ext+" Report.tif");

close("*");

}

//Save results to output directory

saveAs("Results", resultsDir+File.separator+"AnalysisResults.csv");

selectWindow("Log");

saveAs("Text", resultsDir+File.separator+"AnalysisLog.txt");

6. The results, including the final reports and analysis CSV, will be saved in a new folder named *Results_Filtered* within the input directory.

## Data analysis

Manders coefficients depicting the colocalization of the protein of interest can be plotted using GraphPad Prism (RRID:SCR_002798).

## Validation of protocol

This protocol has been used and validated in:

- Tambe et al. [9]. Regulation of OATP1B1 Transport Function by Concurrent Phosphorylation and Lysine-Acetylation-A Novel Post-Translational Regulation Mechanism, Molecular Pharmacology (Figure 8, WT in panels A–C).

**Figure 12.**
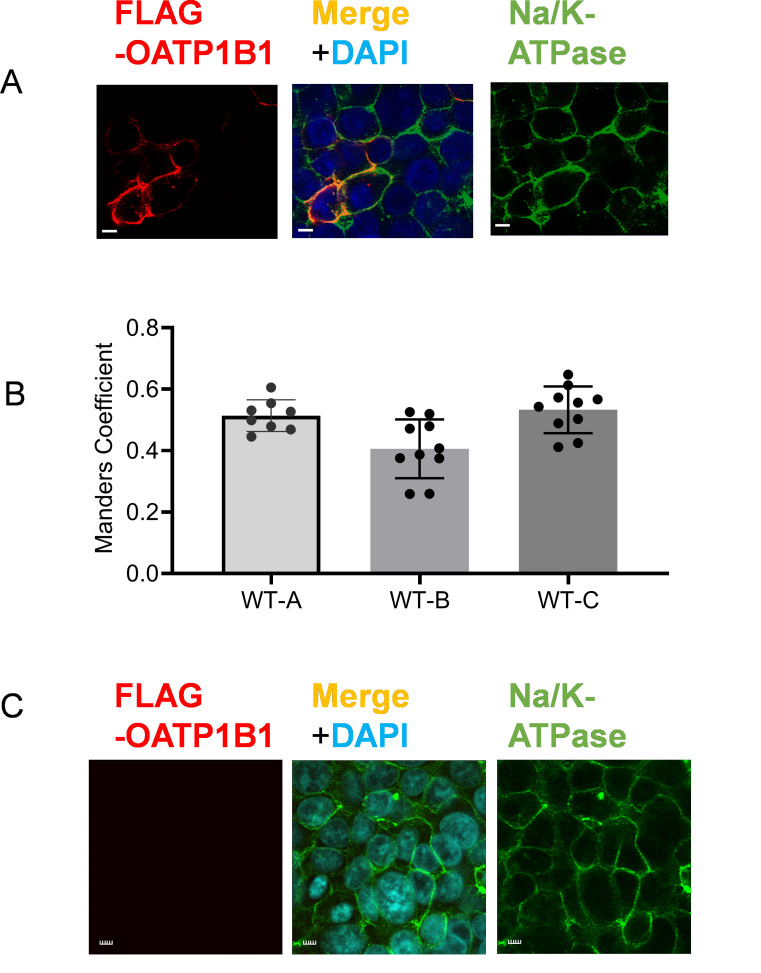
Quantitative colocalization analysis of FLAG-OATP1B1 and Na/K-ATPase in WT-OATP1B1. (A) Co-immunofluorescence staining of FLAG-tagged OATP1B1 (red) and Na/K-ATPase (green) in HEK293 cells 48 h post-transfection with WT-FLAG-OATP1B1. Nuclei were counterstained with DAPI. Representative images are shown. Scale bar, 10 µm. (B) Manders coefficient of colocalization of FLAG-OATP1B1 and Na/K-ATPase. Following the workflow described in this current protocol, confocal images in z-stack were utilized to determine Manders coefficients reflecting the fraction of FLAG-OATP1B1 colocalized with the Na/K-ATPase in WT-OATP1B1 transfected HEK293 cells in Figure 8A–C from [9]. WT-A, WT-B, and WT-C refer to the WT data in Figure 8A, B, and C, respectively, from the original publication [9]. Data represent mean ± SD. Each dot denotes data from replicates of confocal images. Figures are cited and modified from the following reference with permission: Tambe et al. (2025) Molecular Pharmacology, https://doi.org/10.1016/j.molpha.2024.100007 [9]. (C) FLAG and Na/K-ATPase (green) immunofluorescence staining in the negative control, non-transfected HEK293 cells. Nuclei were counterstained with DAPI.

Three images from Figure 5B WT-OATP1B1 of the original publication are reused in Figure 12A of this manuscript. Manders coefficient values of WT-OATP1B1 (WT) in Figure 8A–C of the original publication are replotted here in Figure 12B. The reuse of previous data was approved by the journal.

## General notes and troubleshooting

**General notes**

1. Ensuring consistent confocal image acquisition parameters across all samples in a dataset is crucial for reliable analysis. It is imperative to maintain uniform laser intensity and detector sensitivity settings across a dataset during image capture to facilitate accurate comparisons. Different specimens may respond better to specific thresholding methods; therefore, it is advisable to evaluate a sample image immediately after acquisition to determine the most suitable thresholding method.

**Figure 13.**
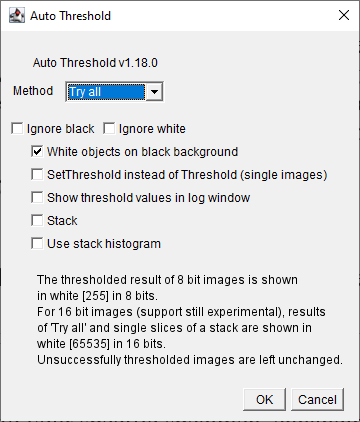
Screenshot of *Auto Threshold* interface. Select *Try all* option from the drop-down menu of *Method*. Check box for *White objects on black background*.

To assess and select the optimal thresholding method in Fiji ImageJ, follow these steps:

a. Launch Fiji ImageJ and load one of your acquired raw images.

b. Navigate to *Image → Adjust → Auto Threshold*.

c. In the dialog box (Figure 13): Select the *Try all* option to apply multiple thresholding algorithms. Check the box labeled *White objects on black background* to ensure proper object-background distinction.

d. Click OK to generate a montage displaying the results of various thresholding methods applied to your image.

e. Review the montage to identify which thresholding method best suits your sample's characteristics. This procedure allows for the selection of the most effective thresholding technique tailored to your specific specimen, enhancing the accuracy of subsequent analyses.

2. Steps to run a macro in Fiji ImageJ:

a. Open *Fiji*.

b. Go to *Plugins → New → Macro.*

c. Copy and paste the macro code from the manuscript into the editor without modifying the indentations. For instance, in a for-loop, all nested commands must retain their original indentation; otherwise, the script may not be executed as intended.

d. Navigate to the menu bar and click on *Run → Run.*

**Discussion**

This protocol significantly reduced the hands-on time required to manually handle large quantities of image files. By automating the data processing, this approach also minimized the risk of human error associated with manual data handling. The protocol can complete the analysis in less than one hour of hands-on time, markedly enhancing efficiency and accuracy. In our current workflow, we utilized the Manders coefficient method and Otsu thresholding. This batch-processing workflow may be further developed and adapted based on this protocol to incorporate other colocalization methods in BIOP-JACoP plugin for determining colocalization. Thus, the protocol has the potential to have a broad impact in the field.

**Troubleshooting**

Problem 1: Out of memory.

Possible cause: Large image files and multiple analyses consume significant RAM, leading to slower performance or errors.

Solution: Optimize Fiji’s memory settings via *Edit → Options → Memory & Threads..*. Allocate sufficient memory based on available computer system resources. For efficient processing, avoid running tasks on networked drives and transfer raw files to a local disk before initiating the workflow.

We successfully processed a 3.89 GB dataset (30 raw Z-stacks) using 12,970 MB of allocated memory in Fiji ImageJ, without encountering memory crashes.

Problem 2: Compatibility issues with plugins or code.

Possible cause: Outdated or incompatible versions of Fiji, BIOP-JACoP, or Python libraries can cause errors.

Solution: Ensure all software and plugins are updated to the specified versions listed in the protocol. For Python scripts, install the required libraries (e.g., pandas) using compatible Python versions (≥3.7). The standalone Python applet is available on GitHub: https://github.com/weiyue99/Yue-Colocalization
